# Recent Advances in Pickering Double Emulsions and Potential Applications in Functional Foods: A Perspective Paper

**DOI:** 10.3390/foods12050992

**Published:** 2023-02-26

**Authors:** Junjia Zhang, Jieyu Zhu, Yujia Cheng, Qingrong Huang

**Affiliations:** Department of Food Science, Rutgers University, 65 Dudley Road, New Brunswick, NJ 08901, USA

**Keywords:** pickering emulsions, double emulsions, colloidal particles, encapsulation, controlled release

## Abstract

Double emulsions are complex emulsion systems with a wide range of applications across different fields, such as pharmaceutics, food and beverage, materials sciences, personal care, and dietary supplements. Conventionally, surfactants are required for the stabilization of double emulsions. However, due to the emerging need for more robust emulsion systems and the growing trends for biocompatible and biodegradable materials, Pickering double emulsions have attracted increasing interest. In comparison to double emulsions stabilized solely by surfactants, Pickering double emulsions possess enhanced stability due to the irreversible adsorption of colloidal particles at the oil/water interface, while adopting desired environmental-friendly properties. Such advantages have made Pickering double emulsions rigid templates for the preparation of various hierarchical structures and as potential encapsulation systems for the delivery of bioactive compounds. This article aims to provide an evaluation of the recent advances in Pickering double emulsions, with a special focus on the colloidal particles employed and the corresponding stabilization strategies. Emphasis is then devoted to the applications of Pickering double emulsions, from encapsulation and co-encapsulation of a wide range of active compounds to templates for the fabrication of hierarchical structures. The tailorable properties and the proposed applications of such hierarchical structures are also discussed. It is hoped that this perspective paper will serve as a useful reference on Pickering double emulsions and will provide insights toward future studies in the fabrication and applications of Pickering double emulsions.

## 1. Introduction

Double emulsions are generally defined as emulsions of emulsions, as they are emulsion systems in which a primary emulsion is subsequently dispersed into the continuous phase of the secondary emulsion. Double emulsions, composed of three phases, are considered to be multiple emulsions with ternary structures [1], though the terms “double emulsion” and “multiple emulsion” are used somewhat interchangeably in practice [2]. Double emulsions are commonly classified into either W/O/W (water-in-oil-in-water) or O/W/O (oil-in-water-in-oil) emulsions. The W/O/W emulsion is composed of a continuous aqueous phase with a dispersed oil phase, which is also the continuous phase encapsulating the innermost aqueous dispersed phase, and vice versa for the O/W/O double emulsion. Double emulsions’ complex structure and multiple interfaces can be denoted as W_1_/O/W_2_ and/or O_1_/W/O_2_ [3] to distinguish their phases and interfaces. However, it is worth mentioning that there have been recent studies exploring the fabrications of water-in-water-in-water (W/W/W) [4,5] and oil-in-oil-in-oil (O/O/O) [6] double emulsions, through methods such as phase inversions, transitions, and utilizations of microfluidic devices.

**Figure 1:**
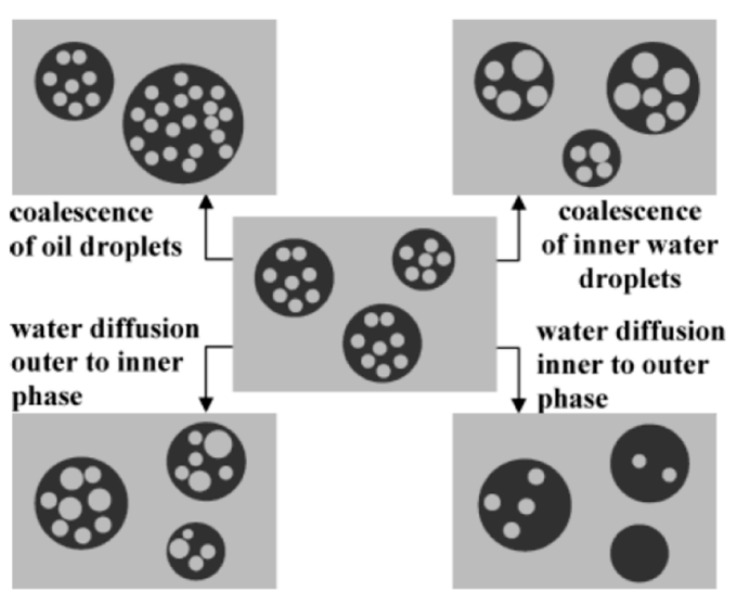
Schematic representation of destabilization routes of conventional double emulsions. Reprinted with permission from [9].

Conventionally, double emulsions are prepared with surfactants as stabilizing agents, while two kinds of surfactants are usually required for the contrasting interfaces [3]. Hence, surfactants of different surface hydrophobicity are used in combination, depending on the surface characteristic (W/O or O/W). However, such double emulsions stabilized solely by surfactants usually lacked kinetic stability [7] and are prone to destabilization because the low molecular weight surfactants can not provide robust interfacial adsorption and can further lead to surfactant migrations between the interfaces. Ultimately, coalescence occurs and induces the collapse of the multiple interfaces, which results in the formation of a simple emulsion [8]. The primary destabilization routes [9] for conventional double emulsions are illustrated in Figure 1. However, other routes of destabilization, such as Ostwald ripening [10], can also threaten the overall stability, especially for polydisperse double emulsions. In addition to the potential instability of double emulsions stabilized solely by surfactants, irritancy [11], environmental concerns [12], and unpleasant sensory effects [2] associated with surfactants further limited their potential towards widespread commercial applications.

Thus, considering the above limitations, double emulsions stabilized by dispersed colloidal particles have gained increased interest. Stabilization of emulsions realized by colloidal particles, also known as Pickering emulsions, was formally acknowledged since the publication of Pickering [13]. Compared to conventional surfactants, interface-adsorbed colloidal particles can establish desorption energy significantly greater than the thermal energy of Brownian motion [14] and can achieve the irreversible adsorption that provides substantial stabilization for double emulsions against coalescence. For instance, the tunable wetting specificity of the colloidal particles enables the stabilization of either the O/W or the W/O interface by adjusting the surface chemistry and/or the hydrophilicity of the particles. The double emulsion is stabilized solely by silica nanoparticles, with tailored particle hydrophobicity to accommodate the multiple interfaces [15]. Moreover, the unique interfacial adsorption properties of the colloidal particles make such double emulsions to be excellent and rigid templates for the fabrication of microspheres [16], microcapsules [17], colloidosomes [18], and other supra-colloidal structures. From now on, this article will refer to double emulsions stabilized by colloidal particles as Pickering double emulsion (PDE). Though it is worth mentioning that the phrases Pickering double emulsion and double Pickering emulsion are used interchangeably among publications. Ideally, the phrase Pickering double emulsion and double Pickering emulsion can be distinguished by double emulsion stabilized by a combination of colloidal particles and co-surfactants, and double emulsion is stabilized solely by colloidal particles. Nonetheless, PDE will be adopted in this article and will include both double emulsions stabilized by a combination of colloidal particles and co-surfactant and by colloidal particles only.

Oza and Frank [19] were recognized as the first to fabricate PDE, where stable W/O/W double emulsion was successfully prepared through a combination of colloidal microcrystalline cellulose and Span surfactant. Since then, clay [20], silica [15,21], metal [22,23,24,25], polymeric [26,27,28], and other colloidal particles have been studied for their potential to stabilize double emulsions. Moreover, colloidal particles obtained from naturally derived substances were also investigated in their potential to stabilize PDE due to the increasing trend for biocompatible and biodegradable materials.

Because of the appealing properties of PDE, the utilization of colloidal particles in the fabrication of double emulsions and their subsequent applications have received considerable interest. Thus, in the hope of providing insights toward future studies in relevant topics, the aim of this paper is to provide perspectives toward advances in PDE over the last decade. The paper will lead with stabilization strategies of PDE and the corresponding colloidal particles. Emphasis is then placed on the proposed and investigated applications of PDE, and the paper concludes with the challenges, limitations, and outlook of PDE.

## 2. Stabilization Strategy

It is worth mentioning that there are two major variations in the number of step(s) of emulsification during the assembly of PDE: the one-step and two-step methods. The two-step method remains the more commonly employed method. It consists of emulsification of a primary emulsion followed by subsequent emulsification into the external dispersed phase to form a secondary emulsion. In contrast, for the one-step method, the emulsification of PDE is achieved in a single homogenization step. Thus, coupled with the two-step method, the combination of hydrophobic and hydrophilic particles with/without co-surfactants is the most prevalent stabilization strategy for the preparation of PDE. As a hydrophilic/hydrophobic particle with/without co-surfactant will be incorporated for stabilization of the primary emulsion for the first step, and another hydrophobic/hydrophilic particle with/without co-surfactant will be utilized for stabilization of the secondary emulsion leading to the formation of the PDE. However, other stabilization strategies were also applied to achieve one-step and/or two-step emulsification, for example, the incorporation of particles with intermediate wettability, environmentally responsive particles, in situ modified particles, and more. The following sections start with an overview of the more conventional stabilization strategy of the combination of hydrophobic and hydrophilic particles for PDE, then move forward to the elaboration of other stabilization strategies. Meanwhile, a summary of the stabilization strategy, the corresponding emulsification steps, colloidal particles utilized, and applications are presented in Table 1.

### 2.1. Combination of Hydrophobic and Hydrophilic Particles with/without Co-Surfactants

#### 2.1.1. Silica Particles

Silica particles are one of the most typical interfacial stabilizers in the preparation of PDE due to their simplicity, wide availability, and versatility. It is found that PDE prepared with silica particles of extreme hydrophilicity and/or hydrophobicity exhibit poor stability against coalescence [15], as colloidal particles should be partially wetted by both the continuous and dispersed silica particles with intermediate hydrophilicity, and hydrophobicity yields improved stability. Moreover, by adsorbing Poly(ethylene imine) (PEI) onto the surface of fumed silica particles, Williams et al. prepared a “hybrid” PEI/silica particle that could stabilize both the W/O and O/W interfaces of PDE through adjustment of the PEI/silica mass ratio [37]. 

A combination of hydrophilic and hydrophobic silica particles is usually required to stabilize the two contrasting interfaces present in PDE [17,29]. In some conditions, hydrophobic/hydrophilic silica particles are used in combination with co-surfactants [30,31], magnetic nanoparticles [16,32,33,65], and polymeric particles [34,35].

#### 2.1.2. Polymeric Particles

Besides silica particles, polymeric particles are also widely used in PDE. Graphene oxide [38], with its high surface-to-volume ratio, has been explored for stabilization of the W_1_/O interface of a W_1_/O/W_2_ PDE by tuning the hydrophilic functional group content through thermal reduction. Hydrophilic and hydrophobic graphene oxide quantum dots [66] were fabricated and employed for the stabilization of W_1_/O/W_2_ PDE. In addition, Thompson et al. [26] prepared hydrophilic and hydrophobic polymer worms through reversible addition–fragmentation chain transfer (RAFT)—mediated polymerization-induced self-assembly and investigated their capability in stabilizing the two interfaces of PDE. Furthermore, Lei et al. [59] has fabricated a high internal phase W/O/W PDE through one-step emulsification by using poly(2-(diethylamino)ethyl methacrylate) (PDEA) microgel particles as a Pickering stabilizer. The team synthesized PDEA microgel particles from 2-(diethylamino)ethyl methacrylate (DEA) monomers and expected the microgel particles to stabilize only the O/W interface, but W/O/W PDEs were achieved in one-step emulsification instead. It was hypothesized that some DEA monomers remained and contributed to the W/O interface stabilization. The combined effect of DEA and PDEA together contributed to the formation of W/O/W PDE.

#### 2.1.3. Metallic Particles 

To endow specific functionality such as electrical, catalytic, and magnetic properties, metallic nanoparticles such as Fe_2_O_3_ [33,67], Fe_3_O_4_ [25,32,39,40,41], and tetra(ethylene glycol), functionalized Au [22] has been utilized for the preparation of PDE. Meanwhile, these colloidal particles are preferentially wetted by the aqueous phase, making them capable of stabilizing the O/W interface and, thus, incorporating additional hydrophobic particles with/without co-surfactant was needed to stabilize the W/O interface of the PDE. Through hydrophobic modification, metallic particles such as oleic acid Fe_2_O_3_ coated particles can also serve as a stabilizer for the W/O interface [33].

#### 2.1.4. Naturally Derived Particles

Due to the increased interest in applications of PDE among pharmaceutical, cosmetic, and food sciences, recent trends in fabricating PDE with naturally derived colloidal particles continue to grow steadily. In the fabrication of W_1_/O/W_2_ PDE, naturally derived colloidal particles such as oligosaccharide particles (e.g., cyclodextrin [49]), polysaccharide particles (e.g., starch and modified starch [42,43,44]); water-insoluble protein particles (e.g., kafirin [45] and zein [46]); fat (e.g., wax [36,56] and mono-and triglyceride crystal [54]); and protein-polysaccharide conjugates (e.g., sugar beet pectin-bovine serum albumin [50] particles) have been used. However, as these particles are preferentially wetted by the aqueous phase, they are prone to stabilizing the O/W_2_ interface of the PDE. Co-surfactants such as PGPR, Span, and lecithin are usually incorporated as well for the stabilization of the W_1_/O interface.

The surface hydrophilicity of some naturally-derived particles can also be easily tuned. For example, soluble complexes and solid particles dried from insoluble complexes [51], obtained from interactions between whey protein concentrate (WPC) and gum Arabic (GA), have been used to stabilize the O_1_/W and W/O_2_ interfaces of O_1_/W/O_2_ PDE. By adjusting the drying temperature during solid particle formation from insoluble WPC-GA complexes, the interface adsorption characteristics of such particles can be further tailored to satisfy the target need [51]. For instance, the fabrication of O_1_/W/O_2_ PDE was also achieved with native and modified nanocellulose particles [52]. The unmodified nanocellulose particles with slight hydrophilicity can stabilize the O_1_/W interface and form a three-dimensional nanofibrils network to protect the emulsion droplets against creaming, while modified nanocellulose particles were able to stabilize the W/O_2_ interface due to the enhanced hydrophobicity introduced from chemical modification with lauroyl chloride [52]. Similarly, hydrophobic and oleic acid-modified lignin, and hydrophilic and unmodified lignin [53] have been combined to stabilize surfactant-free W_1_/O/W_2_ PDE.

Besides proteins and polysaccharides, fats have also been investigated for stabilizing Pickering double emulsions due to their unique capability of crystallization-induced network stabilization and interfacial adsorption as colloidal particles. W_1_/O/W_2_ PDE was prepared by the incorporation of mono- and triglyceride in the primary W_1_/O emulsion and subsequently emulsified into the external sodium caseinate containing the W_2_ phase [54]. It was observed that the fat crystals were capable of adsorbing at the W_1_/O interface by the formation of smooth “shells.” A combination of biopolymers, such as a mixture of gelatin and xanthan gum, and crystallized solid fat was applied to successfully prepare the O_1_/W/O_2_ emulsion [55]. It was postulated that the synergistic effect of interfacial adsorption stabilizes the W/O_2_ interface by crystallized individual fat particles and the physical entrapment of droplets in the network formed by bulk crystallization [55]. Similarly, networked lamellar crystals and a solid layer of adsorbed crystal from carnauba wax [56] have achieved simultaneous stabilization of the O_1_/W and the W/O_2_ interfaces.

Under the aspect of stabilization strategies incorporating naturally derived particles for PDE, especially considering potential applications for edible purposes, gelation of corresponding phases of PDE have also been studied, not only for improving overall stability, but also for lowering the concentration of co-surfactants needed, such as PGPR, which has been reported for irritancy as well as a negative impact on sensory qualities at high concentrations [2]. It has been found that when the external W_2_ phase of the W_1_/O/W_2_ PDE is gelled with an appropriate level of alginate, the storage and encapsulation stability of the PDE is greatly improved [47]. Meanwhile, W_1_/O/W_2_ PDE with beeswax-induced gelation [47,48] of the intermediate oil phase has also demonstrated enhanced stability against processing conditions such as the freeze–thaw cycle and variations in osmotic pressure.

### 2.2. Particles with Intermediate Wettability

**Figure 2:**
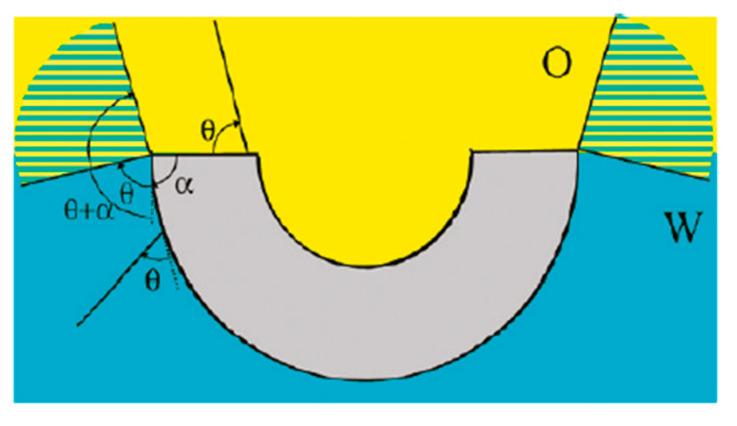
Schematic Illustration of contact angle hysteresis at the surface of microbowl particles. Reprinted with permission from [58].

Besides the hydrophilic and hydrophobic particles discussed above, particles possessing intermediate wettability were also applied in the stabilization of PDE. Heterogeneities in surface properties could be attributed to distinctive particle morphology, as presented in Nonomura, Kobayashi, and Nakagawa’s work, which reported the successful fabrication of PDE stabilized by microbowls [58]. According to the authors’ definition, microbowls are hollowed silica resin particles with holes on the surface. Different PDEs are achieved by combining variously shaped microbowls with oil of different compositions. It is postulated that microbowls demonstrate large contact angle hysteresis due to their distinctive particle shape (Figure 2); the different contact angles caused by microbowls’ morphology enables them to perform not only as a high HLB surfactant, but also as a low HLB surfactant that further allows them to stabilize both outer and inner drop surfaces of the multiple emulsions.

Aside from the influence of particle morphology, various degrees of chemical modification can also generate particles with heterogeneous surface properties. Chen et al. found OS2 particles, a C10-C13 alkylbenzene sulfonic acid hydrophobic-treated boehmite alumina particle with contact angle of about 90°, can establish polydisperse O/W/O PDE in a one-step emulsification process [23]. While claiming OS2 particles as the sole stabilizer of the O/W/O PDEs, the authors also postulated that the OS2 may undergo different degrees of modification and, as a result, exhibit different levels of hydrophobicity: some OS2 particles showed a contact angle slightly larger than 90°, while others have a contact angle equal or less than 90°. Thus, they attributed the formation of PDEs to the stabilization provided by OS2 particles with heterogeneous hydrophobicity. Another similar finding was reported in the work of Bai et al. [60], using surface-modified diatomite particles as the sole stabilizer. Hydrophobic groups were grafted onto diatomite particles by reacting with palmitoyl chloride. It has been speculated that the one-step PDE formation may be attributed to the particle’s heterogeneity in surface wettability, as the surface modification may yield particles with varied amounts of hydrophobic groups and, thus, varied hydrophobicity [60].

### 2.3. Particles with Environmentally Responsive Property

**Figure 3:**
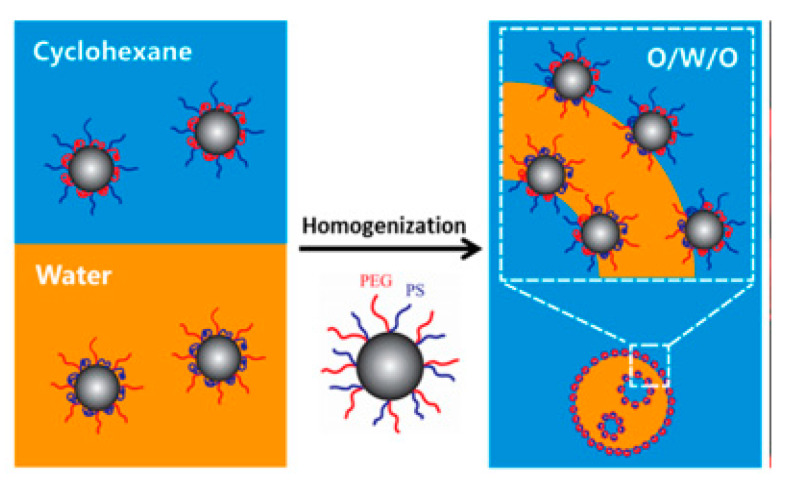
Schematic Illustration of stabilization mechanism of the environmentally responsive terpolymer grafted silica colloidal particles. Reprinted with permission from [11].

Surface modifications have enabled the tailoring of particles that can adjust their surface wettability in response to certain environmental stimuli, such as pH and/or dispersed phase components. These particles, which were regarded as environmentally responsive particles, were also applied in the stabilization of PDE. Due to their flexible surface wettability, such particles can adapt to either the aqueous or oil phases. Therefore, a single kind of particle would be sufficient to accommodate the multiple interfaces of the PDE, which means that it can stabilize either the O/W or W/O interface. For example, amphiphilic silica particles are produced by rafting terpolymers consisting of hydrophilic poly(ethylene glycol) PEG, and hydrophobic polystyrene (PS), and anchoring the block poly[(3-triisopropyloxysilyl) propyl methacrylate] (PIPSMA) onto the particle surface [11]. The terpolymer-grafted silica particles can stabilize either the W/O or the O/W interfaces based on the solvent environment, as if the nanoparticle is dispersed in oil prior to emulsification, the nanoparticle with active hydrophobic PS chains can stabilize the W/O interface and vice versa (Figure 3) [11]. Environmentally responsive particles can also respond to changes in environmental pH. Zhu et al. [28] has synthesized poly(dodecylacrylate-co-acrylic acid) (PDAA) nanoparticles with hydrophobic as well as hydrophilic regions on particle surfaces that can exhibit responsive surface characteristics toward environmental pH. Such polymeric particles can be wetted by both the aqueous and the oil phase under controlled pH value and are, thus, capable of stabilization for both O/W and W/O interfaces.

### 2.4. In Situ Modified Particles

In situ modification is another form of surface modification that can alter the surface properties of a particle. However, in situ modification specifies that particles are modified during the homogenization process by interacting with other components presented in the emulsion precursor system, and the modification site being the interface. Stable PDEs can also be achieved by using colloidal particles that are in situ modified. For example, cross-linked starch nanoparticles (CSTN) were exposed to poly (styrene-co-maleic anhydride) (SMA) which reacted with the hydroxyl group on the surface of CSTN, and consequently rendered the hydrophilic CSTN to be moderately hydrophobic. The in situ modified CSTN can therefore stabilize the W/O interface satisfactorily and can generate stable W/O/W PDEs along with hydroxyethyl cellulose that stabilize the O/W interface [61]. Similarly, the surface wettability of PEI/silica hybrid particles is adjusted by in situ modification to stabilize PDEs [62]. While unmodified PEI/silica particles arre able to stabilize the O/W interface, PEI/silica particles modified by 1-undecanal exhibit enhanced hydrophobicity. Having the W_1_/O interface stabilized by the in situ modified PEI/silica particles and the O/W_2_ interface stabilized by the unmodified PEI/silica particles, stable W/O/W PDEs have been achieved. Zhang et al. [63] also used in situ modified amphiphilic silica particles to achieve W/O/W PDEs through a two-step emulsification process. Primary W/O emulsions were first prepared by using the mixture of styrene (St), tetrathoxysilane (TEOS), hexadecane, and g-(trimethoxysilyl) propylmethacrylate (MPS) as the oil phase, and aqueous triethylamine (TEA) solution as the inner water phase. By the hydrolysis-condensation of TEOS under basic conditions, silica particles were formed and modified by MPS at the O/W interface. Then W/O/W emulsions were fabricated by adding water as the outer phase. The partially modified silica nanoparticles were able to stabilize both the inner and outer droplets of the double emulsions. Aside from adjusting the Pickering particle’s surface wettability, in situ modification can also render stabilizing particles multifunctional. In the work of Ruan et al., corn-peptide-functionalized calcium phosphate (CP-CaP) particles were modified in situ by free fatty acids that presented in the oil phase during the emulsification process [64]. Such modification not only made the CP-CaP particles hydrophobic but also reduced the free fatty acid content in the oil phase, which can contribute to lipid oxidation retardation. The in situ modified CP-CaP particles were able to stabilize both the O/W and W/O interfaces and generate W/O/W PDEs in a sign emulsification step.

## 3. Applications of PDE

### 3.1. Encapsulation of Drugs and/or Nutraceuticals 

Pickering emulsion systems have been studied extensively for encapsulation [68] to protect the encapsulant from degradation or destabilization through direct contact with undesirable environments, such as harsh pH conditions, light, heat, oxidation, and more. In addition, the droplet-in-droplet structure of PDE for enhanced protection had raised promising potentials for them in encapsulation. However, due to the complex nature of PDE, such as its multiple interfaces and the potential diffusion and interactions of encapsulants between phases [57], careful design and preliminary studies are crucial for successful encapsulation and release.

#### 3.1.1. Lipophilic Compounds 

Encapsulation of lipophilic compound within the O_1_ phase of O_1_/W/O_2_ PDE are not as prevalent comparing to encapsulation of water-soluble compound in the W_1_ phase of W_1_/O/W_2_ PDE; Partly due to the reason that oil is not as suitable as water when incorporated as the continuous phase for oral delivery. However, one way to overcome this issue is to remove the outermost O_2_ phase and to use the hierarchical structure with O_1_/W morphology as the encapsulation medium for lipophilic compounds. Thermo-responsive wax-in-water microcapsule fabricated from O/W/O PDE [36] as the shell material, would be broken by expansion of the melting core at 44 °C, has been proposed with the potential of encapsulation of lipophilic compound and heat-stimulated release. However, the thermally induced release could disrupt the heat-sensitive encapsulant prior to target delivery, thus, the physical and chemical properties of the encapsulant should also be evaluated prior to encapsulation into the emulsion matrix.

#### 3.1.2. Hydrophilic Compound 

With carmine encapsulation [42,69] in the W_1_ phase, followed by emulsification, storage, centrifugation release, and determination of carmine present in the W_2_ phase, high encapsulation efficiency (EE) and encapsulation stability (ES) of the Pickering double emulsion has been determined. Similarly, through measurement of the released encapsulant from the innermost to the outermost phase right after emulsification and after a set storage period, W_1_/O/W_2_ PDE also yielded satisfactory EE and ES for encapsulant such as anthocyanin [44,45] and sucrose [43]. The high EE and ES of these PDEs are partially contributed by the robust protection provided by the irreversibly adsorbed colloidal particles, which also prevent coalescence between droplets and diffusion of droplets between phases [69], though other factors, such as osmotic pressure gradient [45] should also be considered for comprehensive assessment in long-term stability.

#### 3.1.3. Co-Encapsulation of Lipophilic and Hydrophilic Compounds

The unique three-phase structure of PDE also gives rise to the potential of co-encapsulation [70]: in the case of W_1_/O/W_2_ PDE, simultaneous encapsulation of hydrophilic and hydrophobic active ingredients in the W_1_ and the O phase has been achieved. With aggregated gliadin nanoparticles adsorbed at the interface and gelatin-induced immobilization of the W_1_ phase [70], enhanced encapsulation stability and 2- and 4-fold increases in the bioaccessibility of W_1_ encapsulated EGCG (epigallocatechin-3-gallate) and O encapsulated quercetin have been obtained. Similarly, betanin has been successfully encapsulated in a gel-like W_1_ phase and curcumin in the oil phase of medium-chain triglyceride, having an encapsulation efficiency of 65.3% and 84.1%, respectively. This PDE co-stabilized by PGPR and sugar beet pectin-bovine serum albumin nanoparticles (SBNPs) was able to prolong the storage stability and bioaccessibility of betanin and curcumin [50]. 

In addition to EE and ES, the digestion and the release profile of the encapsulant are also crucial parameters to be assessed. The digestion profile of PDE could be investigated by simulated digestion coupled with microscopic analysis of the emulsion morphology before and after digestion [46]. For instance, the destabilization mechanism of the colloidal particles and the emulsion matrix and the subsequent release property under digestion are also crucial factors for delivery. As in the investigation of the in vitro digestion profile of a kafirin stabilized W_1_/O/W_2_ Pickering double emulsion [45], though the emulsion system exhibits stable EE and ES during storage for anthocyanin encapsulated in the W_1_ phase, kafirin nanoparticles undergo flocculation and structural collapse during the gastric digestion phase due to the enzymatic digestible property of kafirin. The subsequent emulsion destabilization and release of the droplet contents has been proposed as an outside capsule for target release during the intestinal phase. While W_1_/O/W_2_ Pickering double emulsion stabilized by OSA-modified starch particles [44] has demonstrated the controlled release of W_1_ encapsulated anthocyanin aimed at the intestinal phase, the OSA modified starch-endowed resistance to the acidic and enzymatic condition during the simulated gastric digestion can maintain emulsion integrity for passage to the intestinal digestion.

Regarding the digestion profile of PDE as encapsulation and co-encapsulation delivery systems, recent studies have discovered insightful relationships between tuning the structural properties of the PDE and the corresponding release of the encapsulants. As of PDE’s multi-compartment nature, it was found that when coupled with gelation of selected W_1_, O, and/or W_2_ interfaces of W_1_/O/W_2_, the release profile and the bioaccessibility of the encapsulants can be tuned [47]. More specifically, the release profile and the bioaccessibility of the encapsulant could be manipulated through different combinations of gelation interface(s), as well as the gelation strength. Similarly, incorporations of crystallizable emulsifiers into PDE and the induced crystallization at different interfaces have also been investigated [71]; in which it was found that the site of crystallization played important roles in the rate of structural degradation and lipid digestion of the PDE delivery system, and ultimately offered potentials for tunable release of the encapsulants. 

On top of the contributions toward encapsulation and delivery, manipulations of the structural properties of the PDE could also serve insightful purposes on its sensory profile when considering food and beverage applications. For example, studies [47,48] have found that by gelation of individual phases and/or combination of phases within PDE, as well as controlled gelation strength, the tribological properties of the PDE can be manipulated and result in tunable oral sensation under the aspect of in-mouth smoothness. 

#### 3.1.4. Microbes

PDE had also been adapted for probiotic encapsulation to achieve improved cell viability upon release. Compared to surfactant stabilized double emulsion, the presence of solid particles in the PDE was able to prolong the viability of entrapped cells because the interface-adsorbed particles and the droplet-within-droplet structure of the double emulsion protected the encapsulated microbes from direct contact with the acidic environment during digestion, thereby preventing the rapid loss of viability due to poor tolerance to acidic medium. For instance, β-cyclodextrin-stabilized W_1_/O/W_2_ emulsion had significantly enhanced the cell viability of *Lactobacillus delbrueckii* encapsulated in the W_1_ phase upon release when compared to the surfactant stabilized counterparts [49]. Analogously, *Lactobacillus acidophilus* (LA) encapsulated in the W_1_ phase of a W_1_/O/W_2_ emulsion fabricated with β-cyclodextrin particles yielded improved viability during 14 days storage, and high survival (84%) rate after simulated gastrointestinal digestion in comparison to the free and unencapsulated LA [72]. Furthermore, a 3-fold increase in colon-adhesion efficiency was determined for the W_1_/O/W_2_ encapsulated LA compared to the unencapsulated LA, due to factors such as facilitated adhesion through micelle formation from free fatty acids after oil phase digestion and the gelling effect of sodium alginate incorporated in the W_1_ phase that immobilized the LA [72].

### 3.2. PDE-Templated Hierarchical Structure

#### 3.2.1. Microsphere

Microspheres are spherical particles of diameters within the micrometer range, normally from 1 μm to 1000 μm [73]. Microspheres have a wide range of applications, such as drug encapsulation and delivery [74,75], controlled release [76], catalysis [67], enzyme immobilization [77], sensor [78], and adsorption [41]. Typically, microspheres can be fabricated by solvent evaporation coupled with multiple emulsion templates [79], coacervation methods [80], spray drying [81], polymerization techniques [82], and/or a combination of the above techniques [73,82]. Among the multiple emulsion technique mentioned above, PDE has become an attractive template for the fabrication of microspheres, in contrast to surfactant stabilized double emulsions; because compared to conventional low-molecular-weight surfactants, Pickering stabilizers can assemble at the interfaces without the problems of diffusion between the internal and the external phases, thereby providing a robust template. Moreover, the tunable droplet characteristic allows the fabrication of either monodisperse [27] or polydisperse [16] PDE, in which monodisperse PDE consists of a primary emulsion with a single dispersed droplet, while the polydisperse PDE is made up of a primary emulsion with more than one dispersed droplets. Thus, combined with the advantages mentioned above, PDE is a controllable and versatile template for microsphere fabrication.

The droplet-within-droplet characteristic and the multiple interfaces of PDE also make it ideal for the preparation of microspheres when coupled with polymerization, which is an important and prevalent technique in the fabrication of microspheres. When coupling with the polymerization technique, monomers and initiators can be incorporated into either the O or W phase for W_1_/O/W_2_ [30] or O_1_/W/O_2_ [16] PDE accordingly. Followed by heat, cross-linking, or photo-induced polymerization, the subsequent removal of the external continuous phase will yield the fabricated microsphere. For instance, a hollow microsphere can also be made by the additional removal of the innermost dispersed phase.

**Figure 4:**
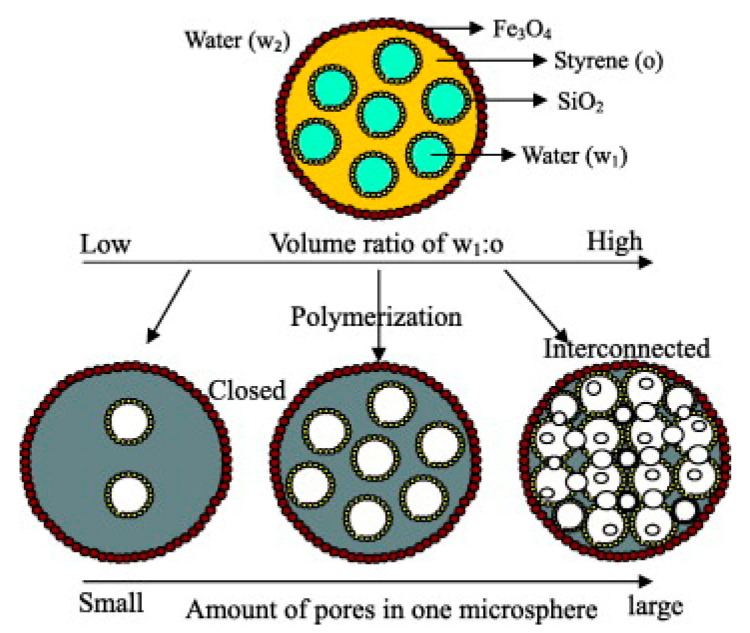
Preparation of multihollow microspheres with tunable pore structures by PDE templating. Reprinted with permission from [32].

One of the advantages of using PDEs as the template for the fabrication of microspheres is that the structure of microspheres can be tailored by adjusting the structure and the property of the PDE. It is found that by varying the volume ratio of the aqueous and the oil phase of the PDE, the pore size and the pore structure can be adjusted for the fabricated porous microsphere (Figure 4) [32,35]. Hu et al. also found that the pore structure of the porous PLGA microsphere can be controlled by variation of the initial PLGA concentration presented in the oil phase of the W_1_/O/W_2_ template PDE [34]. Regulation of the inner structure of the microsphere, from closed-celled to hollowed and to interconnected, can also be achieved by tuning the concentration of the colloidal particles with/without co-surfactants [31]. On the other hand, there has been increased interest in the fabrication of molecularly imprinted microspheres from templating PDE [39,53,66]; for the synthesis of molecularly imprinted microspheres, template molecule and functional monomers are usually incorporated in the O phase of W_1_/O/W_2_ PDE, followed by polymerization and the subsequent removal of the template molecule. Advantages, such as good tunability leading to tailored porosity and the potential of a multi-hollow morphological structure for improved adsorption, have made PDE an attractive template for molecularly imprinted microspheres with versatility.

**Figure 5:**
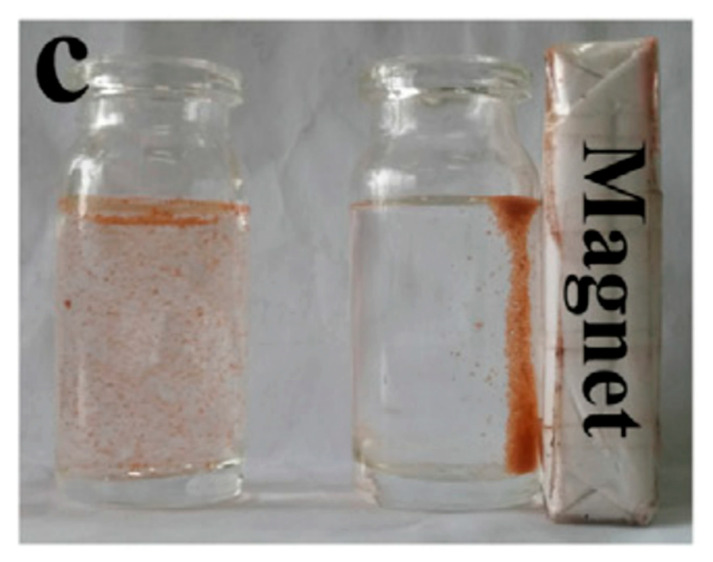
Photograph of magnetic microsphere particles suspended in water and under the presence of an externally placed magnet. Reprinted with permission from [39].

Moreover, colloidal particles presented in PDE can not only serve as an interfacial stabilizer but also endows specific functionalities to microspheres. Magnetic microspheres have been fabricated from PDE by incorporation of interface-adsorbed Fe_3_O_4_ colloidal particles [25,32,41], which provided the microsphere responsiveness to the magnetic stimulus (Figure 5) [39] and has been proposed with applications in the field of carbon fixation, catalysis, heavy metal removal, and wastewater treatment. Ning et al. have further prepared Janus microspheres with dual anisotropy of porosity and magnetism from PDE, with the strategy of polymerization under a magnetic field, as the primary W_1_/O emulsion droplets stabilized by Fe_3_O_4_ particles are concentrated within one side toward the magnetic field [29].

#### 3.2.2. Microcapsule

**Figure 6:**
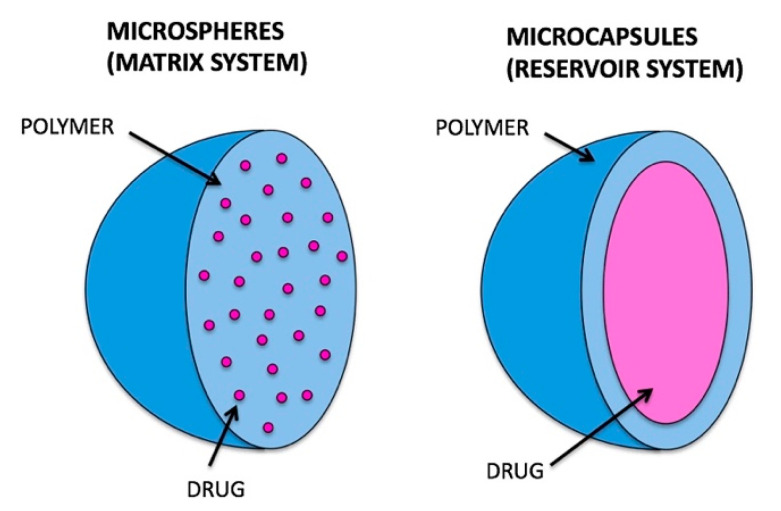
Structure of microspheres (left) and microcapsules(right). Reprinted with permission from [86].

Microencapsulation is the process of encapsulating micron-sized solid, liquid, or gas particles in a shell to protect the encapsulant from the environment [83], in which the microcapsule is one of the vehicles for microencapsulation [84]. Similar to microspheres, microcapsules were defined within the micrometer ranges, and the phrases microcapsule and microsphere were used somewhat analogously in the literature [85]. However, microcapsules and microspheres have different internal structures and morphologies (Figure 6) [84,86]. Different from microspheres, microcapsules possess a distinct core-shell structure with encapsulants containing a core and a surrounding shell built by a layer of polymer(s) and/or solid particles [86]. Because of their distinct core-shell structures, microcapsules are excellent candidates for encapsulation and controlled release of bioactive components [84], masking undesirable attributes such as taste, odor, gastric irritation [87], and more [88]. Techniques to fabricate microcapsules include coating [88], coacervation and phase separation [89], spray drying [90], emulsion and solvent evaporation [84], polymerization [91], and/or a combination of these techniques.

**Figure 7:**
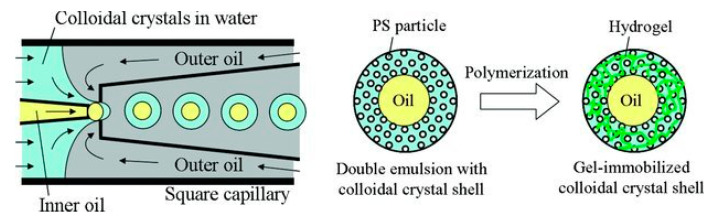
Fabrication of microcapsules with gel-immobilized colloidal crystal shells using capillary microfluidics and photopolymerization. Reprinted with permission from [27].

Thermal responsive microcapsules consisting of a polymeric and colloidal crystal shell, with proposed applications in molecular labeling and biochemical sensors, were synthesized from microfluidic achieved monodispersed O/W/O PDEs via photo-initiated polymerization (Figure 7) [27]. Moreover, wax-in-water microcapsules [36] were assembled from monodispersed O_1_/W/O_2_ PDE, with the silica shell of the microcapsules fabricated from mineralization of surface adsorbed silica particles induced by the mineralization agent tetraethoxy-orthosilane (TEOS). Aside from monodispersed PDE, multi-compartment microcapsules, which enable the co-encapsulation of incompatible compounds for synergistic effects, can be templated from polydisperse PDEs. Multi-compartment microcapsules with a capsule-in-capsule structure [17] were prepared from the templated polydisperse O_1_/W/O_2_ PDE, by in situ polymerizations at both the O_1_/W and the W/O_2_ interfaces coupled with cross-link reaction between the interface adsorbed silica nanoparticles and the shell forming polymers. Two potent chemotherapy drugs, doxorubicin and paclitaxel were loaded in the W_1_ and O phase of a W_1_/O/W_2_ PDE, which was consequently synthesized into core-shell nanocapsule after polymerization with poly(vinyl alcohol) originally present in the O phase [40]. The PVA served the dual purpose of a surfactant as well as shell constituent, whereas the magnetic iron oxide acted as both the shell stabilizer and the trigger for remote drug release under high-frequency magnetic fields. The resulting multi-drug-containing nanocapsules were biocompatible and were proposed for magneto-chemotherapy application [40]. Similarly, W/O/W PDE templating Graphene oxide (GO)@polylactic acid (PLA)@hydroxyapatite (HA) composite microcapsule [38], possessing biocompatible, biodegradable, and pH-sensitive properties has been proposed with potential to load hydrophilic and hydrophobic active compounds simultaneously.

Microcapsules whose shells consist of densely packed colloidal particles are called colloidosomes [18]. Similar to other microcapsules, the sizes and physical properties, such as permeability, mechanical strength, and biocompatibility, of colloidosomes can be precisely tuned through the proper choice of colloids and preparation conditions for their assembly. The high degree of control over their physical properties makes colloidosomes attractive structures for encapsulation and controlled release of active ingredients.

Conventionally, simple W/O Pickering emulsions were commonly adapted to template the colloidal shell structures. However, this approach required subsequent transferring of the colloidosome into a continuous aqueous phase either by centrifugation or repeated washing, which would often induce extra damage to the colloidosome [18]. Compared to simple Pickering emulsions, PDEs have recently been reported to be better templates for colloidosome fabrication, as colloidosomes generated from PDE templates require no further phase transferring and exhibit a narrower range of size distribution. Furthermore, the utilization of PDE as a colloidosome fabrication template also enabled the control over the thickness of the colloidal shell, a vital factor that further affects the permeability and mechanical strength of the colloidosomes by changing the dimension of the PDE templates.

**Figure 8:**
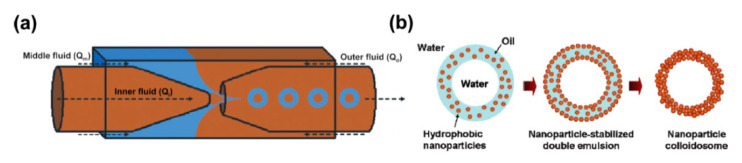
(**a**) Fabrication of PDE via microcapillary device. (**b**) Formation of nanoparticle colloidosomes from W/O/W PDE. Reprinted with permission from [18].

Lee and Weitz were the first to prepare monodisperse semipermeable nanoparticle colloidosomes from W/O/W double emulsion templates generated from glass capillary microfluidic devices (Figure 8a) [18]. Hydrophobic silica (SiO_2_) nanoparticles were dispersed in the oil phase and were reported to adsorb onto the W_1_/O and O/W_2_ interfaces upon the formation of W/O/W PDE, forming colloidal shell structures. The colloidosomes were subsequently fabricated upon removal of the oil phase (Figure 8b) [18]. The colloidosomes-in-colloidosomes structure can also be fabricated from W/O/W PDEs [62]. Hydrophobic and hydrophilic PEI/Silica hybrid particles, obtained by varying PEI surface concentrations of silica nanoparticles, are used to stabilize the W_1_/O interface and O/W_2_ interface, respectively. Cross-linkers were also loaded into the system prior to the emulsification step to provide covalent stabilization, and colloidosomes-in-colloidosomes structures can be achieved through either aqueous phase cross-linking or oil droplet cross-linking, depending on the cross-linker of choice [62].

## 4. Perspectives: Challenges and Outlooks of PDE

Despite the expanding literature on the exploration of colloidal particles for stabilization of the W/O and O/W interfaces of PDE, a majority of the literature has relied on stabilization strategies through a combination of hydrophilic and hydrophobic colloidal particles. However, the incorporation of two kinds of colloidal particles usually requires two separate emulsification steps. Potential destabilization of the primary emulsion might be introduced during the second emulsification step. Furthermore, compared to single-step emulsification, two-step emulsification is more complex, which could limit its scale-up potential for industrial applications. Thus, a potential future research trend lies in developing colloidal particles that could simultaneously stabilize the contrasting interfaces of PDE while achieving emulsification in one step for facile and robust preparation of PDE.

In response to the consumers’ growing demand for organic and clean-labeled products, naturally derived substances have been utilized as a material for food and drug manufacturing. This trend is also reflected in the preparation of PDE, as almost all PDEs stabilized by naturally derived particles were reported in the last ten years. However, PDEs stabilized solely by naturally derived particles were sparse, as co-surfactants are usually required for stabilization of the W/O interface, with PGPR being the most popular option. Despite being excellent stabilizers, most synthetic surfactants have low biocompatibility, are strictly regulated by international regulatory authorities, and can introduce undesirable sensory attributes. Thus, the utilization of hydrophobic naturally derived particles in replacement of synthetic surfactant is preferable in the formulation of PDEs. 

In the future, several potential routes can be investigated to achieve the application potentials of PDE, especially in the field of food, beverage, and nutraceutical sciences: 

(1) Research towards naturally derived particles that can stabilize simple W_1_/O emulsion. 

(2) To tune the surface wettability of hydrophilic naturally derived particles via surface modification methods. Moreover, the current application of PDE in oral delivery focuses primarily on the fabrication of the W/O/W delivery system, while exploration for O/W/O delivery is lacking. This is in alignment with the board range of products that are based on aqueous continuous systems. However, despite being limited, there are popular food products with continuous oil phases, such as butter, migraine, and spreads. More investigations in the preparation of O/W/O PDE for oral delivery could shed light on new formulations of the existing lipid-based food products. 

(3) PDEs are regarded as suitable delivery media for many nutraceuticals and phytochemicals, though PDEs are still rarely adapted as delivery systems for active ingredients due to their poor and uncontrollable stability when exposed to complex human digestive environments. Recent studies are starting to explore the precise tuning of the digestion and release of the encapsulants by engineering the numerous tunable properties of the PDEs. However, more controlled release solutions, such as pH-triggered release, salt-induced release, and temperature-controlled release, could be explored for the PDE as a crucial delivery system to better suit the human digestive system. 

(4) As described in previous sections, hierarchical structures such as microspheres and microcapsules, templated after PDEs, possess desirable characteristics and potentials for applications in terms of controlled release. However, their application in food products is rather limited as non-biocompatible polymers are often required for the polymerization step, which is a critical step to the formation of microstructure. PDE formulated with generally regarded as safe (GRAS) material could potentially give rise to microstructure with biocompatibility. Moreover, the microstructures’ reliance on synthetic polymers could be negated by researching the topic of biopolymerization, which is the polymerization of biopolymers, such as cellulose and wax. If a microcapsule could be templated after GRAS PDE, via bio-polymerization, then its food application could be granted. 

Meanwhile, in many studies, the morphology and configuration of the templating PDE, resulting from adjusting the parameters during preparation of the PDE, have been determined to play critical roles in the microstructure and properties of the fabricated hierarchical structures. However, rather few studies have examined the correlation between variations in the microstructure of the hierarchical structures resulting from tuning the configuration of the templating PDE, and the corresponding changes in the performance of such hierarchical structures in their proposed field of application. Efforts should be made to clarify such correlations and to provide insights for increasing the competency of PDE as a template for the fabrication of hierarchical structures with optimized and tailored performances. For instance, several studies have demonstrated the dual-function role of colloidal particles in PDE, for the cases of using iron oxides as interface adsorbed particles to achieve stabilization of the PDE while providing magnetic property to the fabricated hierarchical structure. As such, the development of nanoparticles responsive to environmental stimuli, such as pH, ionic strength, temperature, mechanical stress, and others, could be another emerging topic seeking to strengthen the versatility of PDE as a template material for hierarchical structures with special purposes. It would then be worthwhile for future studies to venture into exploring colloidal particles that possess dual-function roles and/or environmental responsive properties to provide improved efficacy or increased versatility of the fabricated hierarchical structures based on the templating PDE.

## 5. Conclusions

**Figure 9:**
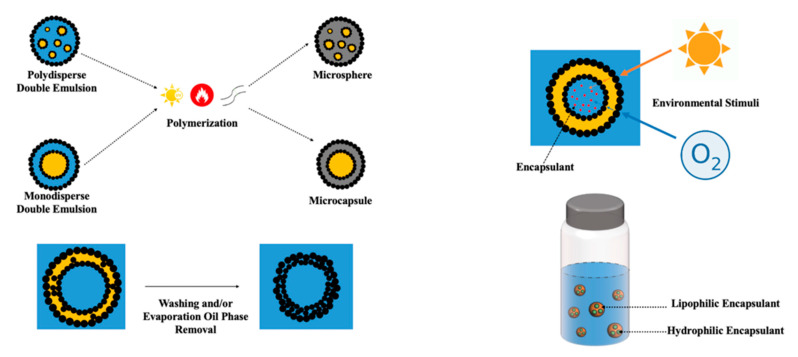
Applications of PDE.

PDE represents a promising alternative to single emulsion and conventional double emulsion stabilized solely by surfactants, with many unique advantages. Compared to a single emulsion, the multi-phases and the consequent multi-compartment properties of PDE allow for more complex tunability and applications, for example, manipulation of the properties of three phases versus two, as well as the potential for co-encapsulation and delivery. While compared to conventional double emulsions, PDE offered enhanced stability and environmental friendliness, meeting satisfaction toward practical purposes. Meanwhile, the rigidity and particle-adsorbed interfaces opened room for PDE to serve as a synthesis template for other hierarchical structures. Consequently, different kinds of colloidal particles and stabilization strategies have been studied extensively. For instance, a broad application prospect has been proposed for PDE; the applications of PDE were outlined in the illustrative Figure 9, from a synthesis template for microspheres, microcapsules, and colloidosomes, to encapsulation with extra layer of protection and co-encapsulation of lipid-soluble and/or water-soluble compounds, which possess promising applications across various fields, such as pharmaceuticals, food and beverage, material sciences, personal care, and dietary supplements. However, it is likely that the potential of PDE has yet to be fully achieved.

## Figures and Tables

**Table 1:** Summary of Stabilizing Strategy, colloidal particles, and applications of PDE.

Emulsification Step	Stabilization Strategy	Colloidal Particle (and Co-Surfactant)	Application	Ref
*Two-Step*	Hydrophobic and hydrophilic particles (co-surfactant)	O_1_/W: Hydrophilic N20 silica W/O_2_: Hydrophobic H30 silica	Synthesis of tunable capsule clusters	[17]
*Two-Step*		O_1_/W: Hydrophilic silica Ludox HS-40 W/O_2_: Hydrophobic silica particle HDK H2000		[21]
*Two-Step*		O_1_/W: Hydrophilic silica particle SLM 1466 W/O_2_: Hydrophobic silica particle SLM1472	Proposed application as an entrapping reservoir for active ingredients	[15]
*Two-Step*		O_1_/W: Hydrophilic G37-H60-B30 block copolymer worms O_1_/W/O_2:_ Hydrophobic L16-B37 Block copolymer worms		[26]
*Two-Step*		W_1_/O: Hydrophobic H30 silica nanoparticle O/W_2_: Hydrophilic mesoporous silica nanoparticles (MSN)	Fabrication of tunable Janus microspheres with magnetism and dual anisotropy of porosity	[29]
*Two-Step*		W_1_/O: Lecithin and hydrophobic AEROSIL R974 silica O/W_2:_ Hydrophilic HKD N20 silica nanoparticle	Preparation of high internal phase Pickering double emulsions	[30]
*Two-Step*		W_1_/O: Span 80 and hydrophobic SiO_2_ O/W_2:_ Hydrophilic SiO_2_	Fabrication of multi-hollow microspheres	[31]
*Two-Step*		O_1_/W: Laponite RD clay W/O_2_: Hydrophobic modified H30 silica particle	Suspension polymerization template for synthesis of multi-hollow polymer microspheres	[16]
*Two-Step*		O_1_/W: Fe_2_O_3_ nanoparticle and Laponite RD clay W/O_2_: H30 silica particle and oleic acid coated Fe_2_O_3_ nanoparticle	Proposed application as polymerization vessel to fabricate nanocomposite polymer microspheres	[32]
*Two-Step*		W_1_/O: Hydrophilic H30 silica nanoparticle O/W_2:_ P(NIPAm-co-MAA) microgels	Stimulus-responsive (pH/temp) emulsion for controlled release.	[33]
*One-Step*		W_1_/O/W_2:_ Hydrophilic SiO_2_ nanoparticle and PLGA	Fabrication of microporous microsphere	[34]
*Two-Step*		W_1_/O: Modified hydrophobic SiO_2_ particle O/W_2:_ PVA	Fabrication of microsphere with aqueous core	[35]
*Two-Step*		O_1_/W: Modified A380 silica and CTAB-functionalized silica W/O_2_: Down Corning surfactant DC3225C	Fabrication of wax–water–SiO_2_ microcapsule with thermo-stimulable release property	[36]
*Two-Step*		W_1_/O: Hydrophobic silica/PEI hybrid nanoparticles O/W_2:_ hydrophilic silica/PEI hybrid nanoparticles		[37]
*Two-Step*		W_1_/O: Graphene oxide (GO) nanoparticle O/W_2_: Hydroxyapatite (HA) nanoparticle	Fabrication of multi-drug containing composite microcapsule for controlled release	[38]
*Two-Step*		O_1_/W: PEI and Fe_3_O_4_ nanoparticle W/O_2_: SM-CaCO_3_ nanoparticle	Template for polymerization-induced synthesis of magnetic Fe_3_O_4_/polyamine hybrid microsphere	[25]
*Two-Step*		W_1_/O: Fe_3_O_4_ nanoparticle O/W_2_: Cellulose nanocrystals	Fabrication of multihollow magnetic imprinted microspheres	[39]
*One-Step*		W_1_/O/W_2_: PVA and iron oxide nanoparticles	Synthesis of multi-drug encapsulated nanocapsules with magnetism	[40]
*Two-Step*		O_1_/W: APTMS coated Fe_3_O_4_ nanoparticle and pluronic F68 W/O_2_: PGPR	Synthesis of magnetic porous microspheres for absorption	[41]
*Two-Step*		O_1_/W: Au-TEG nanoparticle W/O_2_: CdSe QDs		[22]
*Two-Step*		W_1_/O: PGPR O/W_2_: Modified quinoa starch		[42]
*Two-Step*		W_1_/O: PGPR O/W_2_: Waxy starch	Encapsulation of sucrose to enhance sweetness perception	[43]
*Two-Step*		W_1_/O: PGPR O/W_2_: Octenylsuccinate quinoa starch	Encapsulation of anthocyanin	[44]
*Two-Step*		W_1_/O: PGPR O/W_2_: Kafirin	Encapsulation of anthocyanin	[45]
*Two-Step*		W_1_/O: Lecithin O/W_2_: Zein nanoparticles		[46]
*Two-Step*		W_1_/O: PGPR O/W_2_: Bacterial cellulose	Co-encapsulation and controlled release	[47,48]
*Two-Step*		W_1_/O: Span-80 /O/W_2_: β-cyclodextrin	Encapsulation of *Lactobacillus dellbrueckii*	[49]
*Two-Step*		W_1_/O: PGPR O/W_2_: Sugar beet pectin–bovine serum albumin nanoparticles	Co-encapsulation of betanin and curcumin	[50]
*Two-Step*		O_1_/W: Soluble WPC-GA complex W/O_2_: Hydrophobic particles obtained from insoluble WPC-GA complexes		[51]
*Two-Step*		O_1_/W: Nano-fibrillated cellulose (NFC)/Sulfated cellulose nanocrystals (CNC) W/O_2_: Modified NFC/modified CNC	Proposed applications in the fields of food, pharmaceuticals, and cosmetics	[52]
*Two-Step*		W_1_/O: lipophilic lignin O/W_2_: hydrophilic lignin	Fabrication of molecularly imprinted multi-hollow microspheres	[53]
*Two-Step*		W_1_/O: fat crystals O/W_2_: Sodium caseinate		[54]
*Two-Step*		O_1/_W: Gelatin and xanthan gum W/O_2_: Vegetable fat crystal	Proposed for low-fat formulation for margarines and spreads	[55]
*One-Step*		O_1/_W/O_2_: Carnauba wax		[56]
*Two-step*		O_1_/W: Cyclodextrin W/O_2_: Candelilla wax		[57]
*One-Step*		O_1_/W/O_2_: Microbowls	Template to obtain supracolloidal systems	[58]
*One-Step*		O_1_/W/O_2_: Modified Boehmite alumina particles	Controlled release for pharmaceutical field	[23]
*One-Step*		W_1_/O/W_2_: PDEA microgel particle		[59]
*One-Step*		O_1_/W/O_2_: Palmitoyl chloride-modified diatomite particles	Synthesis of porous polyacrylamide particles	[60]
*One-Step*	Environmentally responsive particles	O_1_/W/O_2_: Terpolymer-grafted silica nanoparticles	pH-dependent controlled release	[11]
*One-Step*		O_1_/W/O_2_: PDAA nanoparticle		[28]
*Two-Step*	In situ modified particles	W_1_/O: In situ modified cross-linked starch nanoparticles O/W_2_: Hydroxyethyl cellulose		[61]
*Two-Step*		W_1_/O: in situ modified PEI/silica particles O/W_2_: PEI/silica particles	Fabrication of colloidosomes-in-colloidosomes structure	[62]
*Two-Step*		W_1_/O: In situ modified amphiphilic silica O/W_2_: In situ modified amphiphilic silica	Fabrication of hollowed microspheres	[63]
*One-Step*		W_1_/O/W_2_: In situ modified corn-peptide-functionalized calcium phosphate	Free fatty acid scavenging and lipid oxidation retardation	[64]

## Data Availability

Data is contained within the article.
